# First Decennial Catalogue of College of Physicians and Surgeons of Chicago

**Published:** 1892-07

**Authors:** 


### BOOK NOTICES.

FIRST DECENNIAL CATALOGUE OF THE COLLEGE OF PHYSICIANS AND SURGEONS OF CHICAGO, 1881 TO 1891, AND THE ANNOUNCEMENT FOR 1892 AND 1893. Chicago: Published by the College (813 West Harrison Street), 1892.

The college of which this book is the catalogue and announcement belongs to the *regular* school of medicine. It is located in Chicago directly opposite the Cook County Hospital.

The requirements for admission to this college are: (1) A credible certificate of good moral character. (2) A recommendation from a physician of good standing who is willing to act as the student's preceptor. (3) A diploma from a literary or scientific school granting the degree of B. S. or B. A., or their equivalent; a diploma from a State normal school or from a high school, which will admit to the State University of the State in which the high school is located or other satisfactory certificates. Failing these the student must undergo a prelimary examination, which is detailed in the catalogue. This course of study covers four years. Copies of this book may be had upon application to Dr. Bayard Holmes, Secretary, 240 Wabash Avenue, Chicago.

